# High *Yersinia* prevalence in tonsils of wild boars hunted in Northeast Germany using a novel protocol including long cold enrichment

**DOI:** 10.1128/spectrum.00934-26

**Published:** 2026-07-02

**Authors:** Jens A. Hammerl, Andrea Barac, Nertilda Cako, Sarina Hilke, Claudia Jäckel, Diana Manta, Mariana Marggraf, Martin Richter, Stefan Hertwig

**Affiliations:** 1 Consiliary Laboratory for Yersinia in Food, Department of Biological Safety, German Federal Institute for Risk Assessment 27652, Berlin, Germany; University of São Paulo, São Paulo, Brazil

**Keywords:** *Yersinia *sp., BT 1A, cultural detection, cold enrichment, prevalence, tonsils, wild boars

## Abstract

In this study, the prevalence of *Yersinia* in wild boars in Northeast Germany was investigated. To also detect small numbers of the bacteria by the help of cold enrichment, tonsils of 81 wild boars hunted in 2023/2024 were stored for 12 weeks at 4°C, followed by freezing until usage. The presence of *Yersinia* in thawed tonsils was analyzed by cultural detection using a method based on a previously published protocol, including filtration and centrifugation steps and a selective enrichment for 6 h. *Yersinia* could be isolated from every animal. *Yersinia enterocolitica* and *Yersinia pseudotuberculosis* were recovered from 96% and 21% of the tonsils, respectively. In addition, we isolated eight other *Yersinia* species: *Y. aleksiciae*, *Y. bercovieri*, *Y. frederiksenii*, *Y. intermedia*, *Y. kristensenii*, *Y. massiliensis*, *Y. mollaretii*, and *Y. rohdei*; the most prevalent of those was *Y. kristensenii* (41.9%). A total of 1,105 *Y. enterocolitica* isolates (1–116 per sample) were examined by multiplex PCR, revealing that two tonsils contained pathogenic *Y. enterocolitica* 1B/O:8, whereas all other isolates belonged to biotype (BT) 1A. One hundred eleven isolates were sequenced. The enterotoxin gene *ystB* was detected in every BT 1A isolate, while some of them additionally possessed the virulence genes *ail*, *myfA*, and *tccC. Y. pseudotuberculosis* isolates belonged to BT 1 or 2 and either to the sero/sequence type O:1a/42, O:1/43, or O:2a/14. The study suggests that wild boars are a natural reservoir for *Yersinia* and that the animals may be involved in their spread and in the contamination of vegetables like carrots and salad.

**IMPORTANCE:**

*Yersinia enterocolitica* and *Yersinia pseudotuberculosis* are zoonotic agents, which may infect humans and a broad range of animals. PCR analyses suggest that these species are widespread in the environment, even though exact prevalence data are rare due to the fact that the isolation of the bacteria is still challenging. We therefore developed a protocol, which allows a sensitive and quick cultural detection of *Yersinia* in meat samples. In this study, the protocol was used in conjunction with a long cold enrichment for the isolation of *Yersinia* from tonsils of wild boars. It turned out that wild boars indeed are carriers for numerous *Yersinia* species. Of great significance here is a high prevalence of *Y. pseudotuberculosis*. Since the bacteria occur in both the tonsils and the gut, it cannot be excluded that they may be transmitted by wild boars to other animals and to plants through direct contact or fecal shedding.

## INTRODUCTION

*Yersinia enterocolitica* and *Yersinia pseudotuberculosis* are enteropathogenic species causing diseases termed yersiniosis (1, 2). Yersiniosis is the third most common bacterial enteritis in Europe, with 3,825 reported cases for Germany in 2024 (SurvStat@RKI, v2.0, https://survstat.rki.de/Default.aspx). Typical symptoms are diarrhea, abdominal pain, and fever, which occur mostly in young children (3). The disease is mainly caused by *Y. enterocolitica*, whereas only a few cases of human yersiniosis have been reported for *Y. pseudotuberculosis*. Reasons for this may be the difficult cultural detection of this species and different reservoirs of *Y. enterocolitica* and *Y. pseudotuberculosis*. The presence of *Y. enterocolitica* is closely associated with pigs, to which this species is well adapted (4). The bacteria can be found in feces and particularly in tonsils, from which contaminations may occur during slaughtering (5). Infections are mainly caused by the consumption of raw or undercooked pork (6–12). The species comprises six biotypes (BTs) (1A, 1B, 2, 3, 4, and 5) and approximately 70 serotypes, not all of which are pathogenic (13). While bio/serotype 1B/O:8 strains harboring the 70 kb virulence plasmid pYV and a pathogenicity island are highly virulent, those belonging to BT 1A do not contain pYV and several chromosomal virulence genes existing in the other BTs. They have long been considered non-pathogenic, even though there is an increasing number of reports describing BT 1A isolates from clinical cases (14–22). In Switzerland, for instance, 34% of 149 *Y. enterocolitica* isolates associated with human infections belonged to BT 1A and revealed high diversity (23). It has been reported that BT 1A comprises at least two phylogenetic lineages, each with different virulence factors, e.g., toxins (24, 25). By contrast, even though *Y. pseudotuberculosis* may also be composed of isolates exhibiting diverging virulence, it does not show such heterogeneity like *Y. enterocolitica* but has a more diverse host spectrum. It is not only a human pathogen but can also infect livestock and pets, as well as wild and zoo animals, with sometimes lethal outcomes (26–30). As the demand for foods from unconventional production (e.g., game meat or outdoor farming of domestic pigs) is constantly increasing, pathogenic *Yersinia* species may gain greater importance as foodborne pathogens through the consumption of, e.g., wild boar meat and products made from it. During the hunting season 2023/2024, 542,468 wild boars were shot in Germany, 15% more than in the season before. Nonetheless, there are currently only a few data available on the prevalence of *Yersinia* in game. Most studies were carried out in Europe. In Italy, various samples (feces, liver, meat, mesenteric lymph nodes, spleen, and swabs taken from carcasses and tonsils) of wild boars were examined by cultivation and/or PCR for the presence of *Yersinia* (31–36). *Yersinia enterocolitica*, of which most isolates belonged to BT 1A, was detected in up to 30.3% of the tested animals by culturing and up to 75% by PCR. In addition, other non-pathogenic *Yersinia* species (*Y. aldovae*, *Y. bercovieri*, *Y. frederiksenii*, and *Y. intermedia*) were isolated, while *Y. pseudotuberculosis* could only rarely be recovered. Similar data were reported in Finland, Poland, and Switzerland (37–40). In Germany, 111 tonsils of wild boars were examined for *Y. enterocolitica*, which was isolated with a prevalence of 17.1% (41). Here, all but two isolates belonged to BT 1A. Another study in Germany focused on *Y. pseudotuberculosis*. Only 10 out of 503 tested tonsils (2%) were positive for this species by cultivation (42, 43). However, 6.4% of the tonsils were positive by PCR, indicating a higher prevalence. In fact, seroprevalence data of *Yersinia* suggest even higher numbers of infection (44–46). Publications on this subject also demonstrated that enteropathogenic *Yersinia*, especially *Y. enterocolitica*, can more efficiently be isolated from tonsils than from feces of pigs and that cold enrichment for up to 28 days at 4°C in PSB broth gave better results than selective enrichment in Irgasan ticarcillin chlorate broth for 48 h (37, 39, 47–50). The ISO 10273:2017 method (https://www.iso.org/standard/63329.html) of the European Committee for Standardization, which is often used for such studies, aims mainly at the cultural detection of pathogenic *Y. enterocolitica* from food. We have recently optimized the method by introducing a filtration and centrifugation step in combination with enrichment for only 6 h, allowing a sensitive cultural detection of *Yersinia* within 1 day and a limitation of background bacteria (51). In this study, this method was used for the isolation of yersiniae from wild boar tonsils after storage of the tonsils for 12 weeks at 4°C to find out whether an extended enrichment at this temperature may further increase the cultural detection of the bacteria.

## RESULTS

### High prevalence of yersiniae in the investigated wild boars

*Yersinia* spp. could be isolated from all 81 animals. Altogether, 10 different *Yersinia* species were recovered; the most predominant was *Y. enterocolitica*, which was isolated from 78/81 (96.3%) of the tonsils (Table 1 and Table S1). On the other hand, *Y. pseudotuberculosis* was isolated from 17/81 (21%) of the samples. Besides these two pathogenic species, eight non-pathogenic *Yersinia* species were isolated. The highest prevalence here was determined for *Y. kristensenii* with 41.9%. The remaining species were isolated from 1 to 14 animals. A total of 1,455 colonies displaying a bull’s-eye morphology that were picked from CIN agar plates were identified as *Yersinia* sp. by matrix-assisted laser desorption ionization–time of flight mass spectrometry (MALDI-TOF MS). The microbial background flora on CIN comprised mainly the genera *Pseudomonas* and *Serratia*, which showed a similar colony morphology. In contrast to *Yersinia*, in total only 209 and 102 colonies belonging to *Serratia* and *Pseudomonas*, respectively, were isolated (Tabe S2). Thus, *Yersinia* was by far the prevailing genus.

**TABLE 1:** Occurrence of *Yersinia* species in wild boars hunted in different counties of the federal state of Brandenburg, Germany

District	*Y. enterocolitica*	*Y. pseudotuberculosis*	*Y. frederiksenii*	*Y. kristensenii*	*Y. rohdei*	*Y. massiliensis*	*Y. intermedia*	*Y. bercovieri*	*Y. mollareti*	*Y. aleksiciae*
Dahme-Spreewald	15/15 (100%)	5/15 (33.3%)	4/15 (26.7%)	7/15 (46.7%)	2/15 (13.3%)	6/15 (40%)	2/15 (13.3%)	1/15 (6.7%)	0/15 (n.d.)	0/15 (n.d.)
Havelland	31/33 (93.9%)	5/33 (15.2%)	4/33 (12.1%)	11/33 (33.3%)	0/33 (n.d.)	6/33 (18.2%)	4/33 (12.1%)	4/33 (12.1%)	1/33 (3.0%)	1/33 (3.0%)
Märkisch Oderland	4/5 (80%)	2/5 (40%)	1/5 (20%)	2/5 (40%)	0/5 (n.d.)	0/5 (n.d.)	0/5 (n.d.)	1/5 (20%)	0/5 (n.d.)	0/5 (n.d.)
Oberhavel	16/16 (100%)	3/16 (18.8%)	1/16 (6.3%)	6/16 (37.5%)	0/16 (n.d.)	2/16 (12.5%)	2/16 (12.5%)	1/16 (6.3%)	0/16 (n.d.)	0/16 (n.d.)
Ostprignitz Ruppin	6/6 (100%)	0/6 (n.d.)	0/6 (n.d.)	4/6 (66.7%)	0/6 (n.d.)	0/6 (n.d.)	1/6 (16.7%)	1/6 (16.7%)	1/6 (16.7%)	0/6 (n.d.)
Uckermark	6/6 (100%)	2/6 (33.3%)	1/6 (16.7%)	4/6 (66.7%)	0/6 (n.d.)	0/6 (n.d.)	2/6 (33.3%)	1/6 (16.7%)	0/6 (n.d.)	0/6 (n.d.)
Overall	96.3% (78/81)	20.9% (17/81)	13.6% (11/81)	42.0% (34/81)	2.5% (2/81)	17.3% (14/81)	13.6% (11/81)	11.1% (9/81)	2.5% (2/81)	1.2% (1/81)

**Fig 1:**
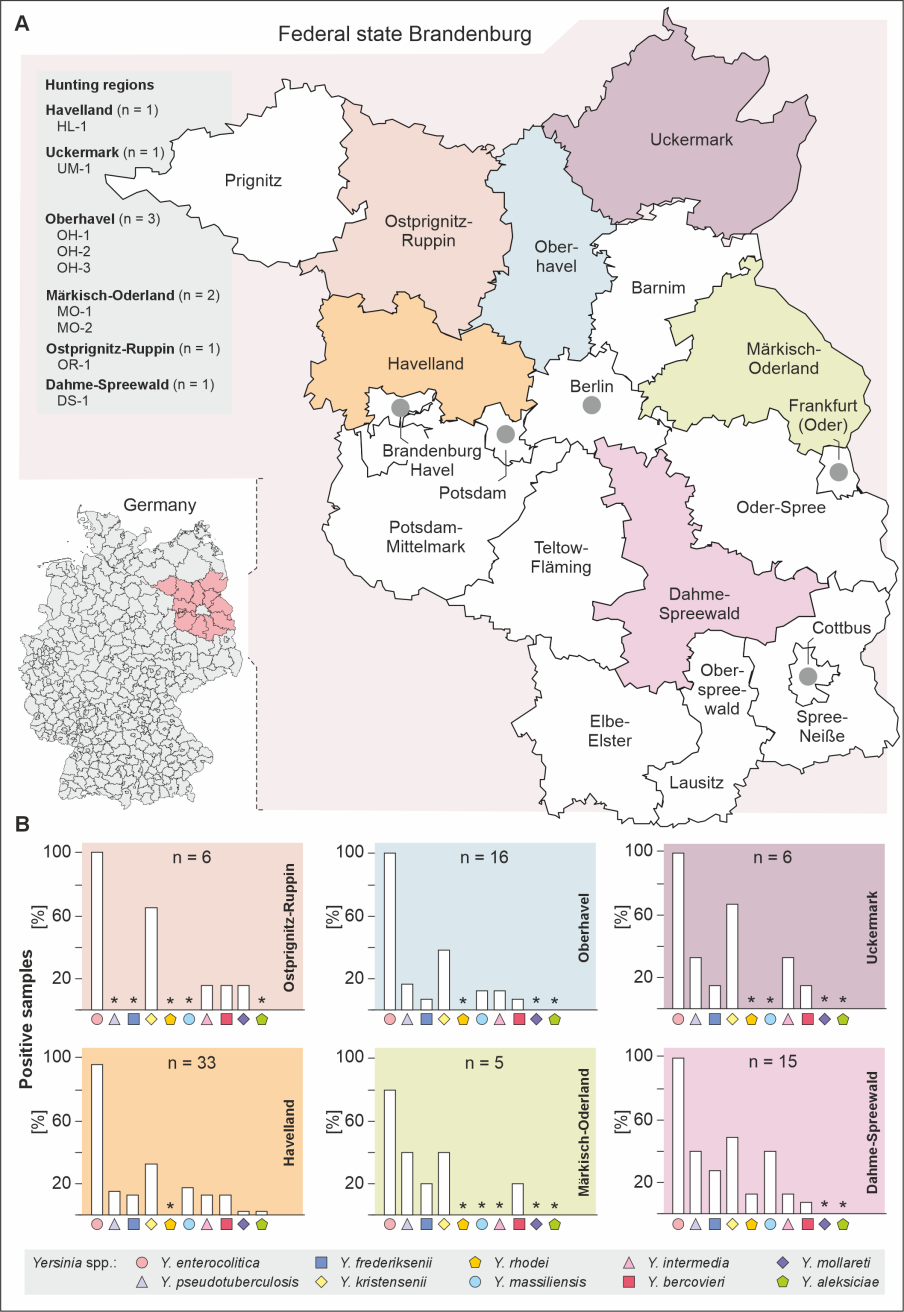
Prevalence of *Yersinia* spp. in samples from wild boars in different counties of the German federal state of Brandenburg. (**A**) Location of the hunting grounds. The map was created using ArcGIS software v10.2.2, with a base map from the Federal Agency for Cartography and Geodesy (Datenlizenz Deutschland, Namensnennung, v2.0; www.govdata.de/dl-de/by-2-0). (**B**) *Yersinia* species isolated from tonsils in each county. The use of colors and symbols is specified in the gray box. White bars indicate the percentage of samples that tested positive for the respective *Yersinia* species. Asterisks indicate that the species was not detected.

Regarding the age and sex of the animals, no significant tendencies were apparent (Table S3). However, while *Y. enterocolitica* was isolated from almost all animals shot in the different counties of Brandenburg, this was not the case with *Y. pseudotuberculosis*. Here, significant differences were observed, with prevalences between 0% (Ostprignitz Ruppin) and 40% (Dahme Spreewald and Märkisch Oderland) (Fig. 1B, Table 1 and Table S1). Similar variations were obtained with some *Y. enterocolitica*-like species, e.g., *Y. intermedia* and *Y. massiliensis*, while *Y. kristensenii* was isolated in each county with prevalences between 33.3% and 66.7%.

### Most isolates belong to *Yersinia enterocolitica* BT 1A

**Fig 2:**
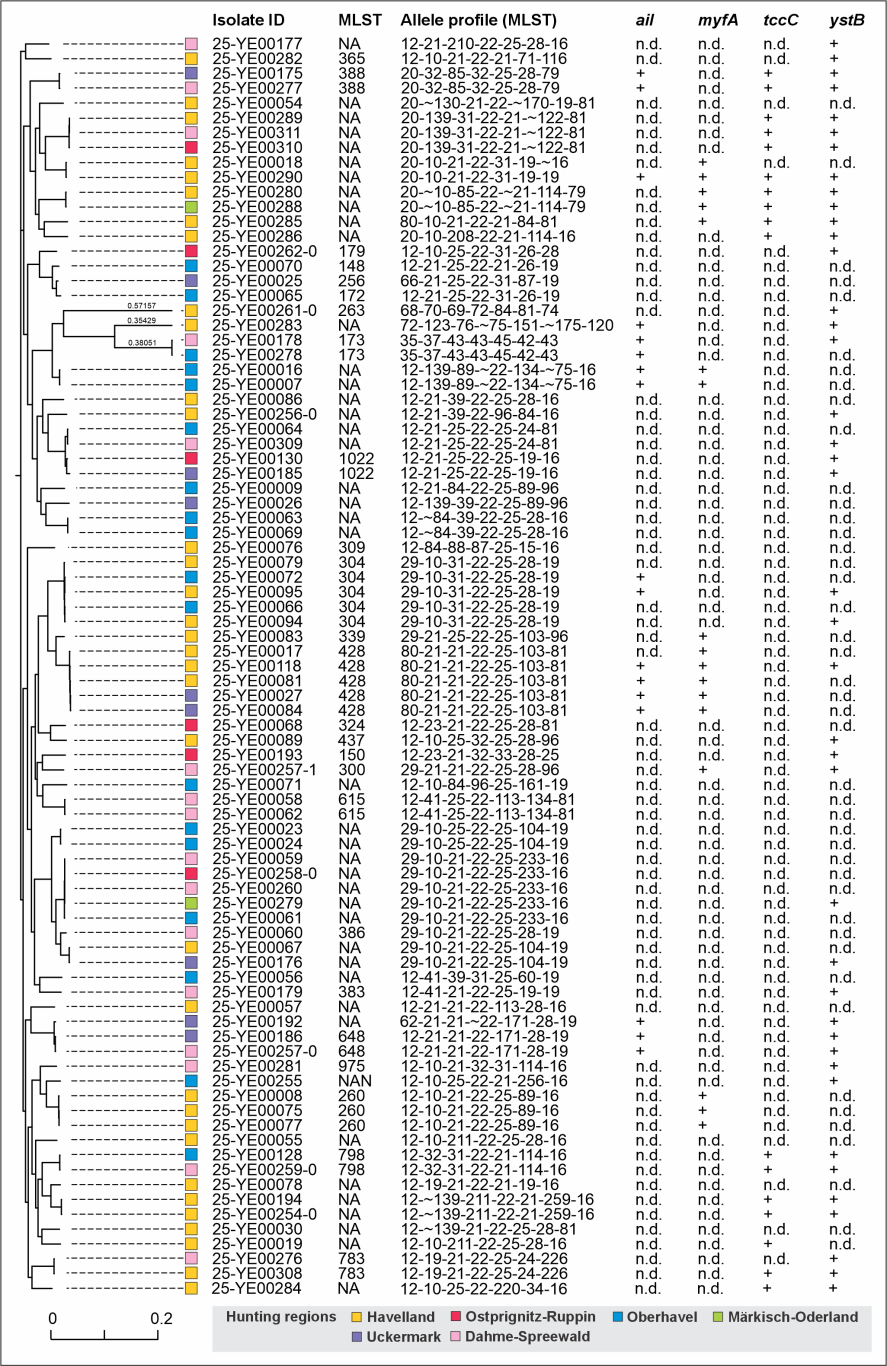
Phylogenetic relationship of *Y. enterocolitica* isolates. Isolate 25-YE00011 has been used as a reference for phylogenetic comparison. All *Y. enterocolitica* isolates covered 73.88% of the reference genome (genome size: 4,760,776 nt). A standard for substitution per site is given. The ST types and the presence of some virulence-associated genes are indicated. Abbreviations: NA, not available; n.d., not detected.

A total of 1,105 isolates identified as *Y. enterocolitica* by mass spectrometry were subjected to a multiplex PCR, allowing the detection of both pathogenic and non-pathogenic BTs (52). Besides two of them, which belonged to the highly virulent bio/serotype 1B/O:8 containing pYV, all other isolates were positive for the genes *gluF* and *ystB*, typical for BT 1A. Some isolates were additionally *ail* positive (Fig. S1). To study the heterogeneity of BT 1A, at least one isolate from each tonsil was sequenced. Bioinformatic analysis of whole-genome sequencing (WGS) data revealed that the tonsils harbored isolates belonging to numerous sequence types (STs) (Fig. 2 and Table S4). Altogether, out of 84 sequenced BT 1A genomes, 54 different MLST allele profiles (*adk-argA-aroA-glnA-thrA-tmk-trpE*) were determined, of which only 25 matched with as-yet-unassigned STs. The most frequent ST was ST304 (*n* = 5). Phylogenetically, the genomes showed great genetic diversity, similar to the sequence types. A search for virulence-associated genes showed that all isolates contained the genes *hreP*, *invA*, *yaxA*, *yaxB*, and *ystB*, as well as the complete *fep-fes* enterochelin gene cluster for siderophore uptake (53). In some isolates, the genes *ail*, *myfA*, and *tccC*, the latter of which is part of the insecticidal toxin complex TC (54), were additionally identified (Fig. 2 and Table S4 ). However, while *tccC* showed strong identities (≥95%) to the corresponding gene in the database, other genes (*tcbA* and *tcaC*) of the complex were much more dissimilar. The strong diversity of the BT 1A isolates was also reflected by their plasmid content. *In silico* analyses indicated that 50 out of 84 genomes, contigs match fully or in part with plasmids of the public databases, leading to cumulative plasmid sizes ranging from 3 to 238 kb. Contigs that shared similarities with up to four different plasmids were detected in some isolates. It was conspicuous that very similar or even identical plasmids were predicted for several isolates, regardless of their origin (date and place of hunting). However, in 33 of the 50 genomes, none of the yet assigned plasmid replicons (incompatibility [Inc]) types were identified, suggesting the presence of yet unknown incompatibility groups or mobile genetic elements (e.g., insertion sequences and conjugative islands) integrated into the chromosome. Among *Y. enterocolitica* genomes that exhibited Inc types, IncFII and IncX (X1 and X4) replicons were identified (Table S4). High sequence similarities were found in plasmid sequences of *Y. massiliensis* strain GTA 1 and *Y. enterocolitica* strain Y201. However, certain other plasmids of *Escherichia coli*, *Salmonella*, *Serratia*, and *Citrobacter* also partially match the presumptive plasmid contigs determined by *in silico* analysis (data not shown).

### *Yersinia pseudotuberculosis* isolates

**Fig 3:**
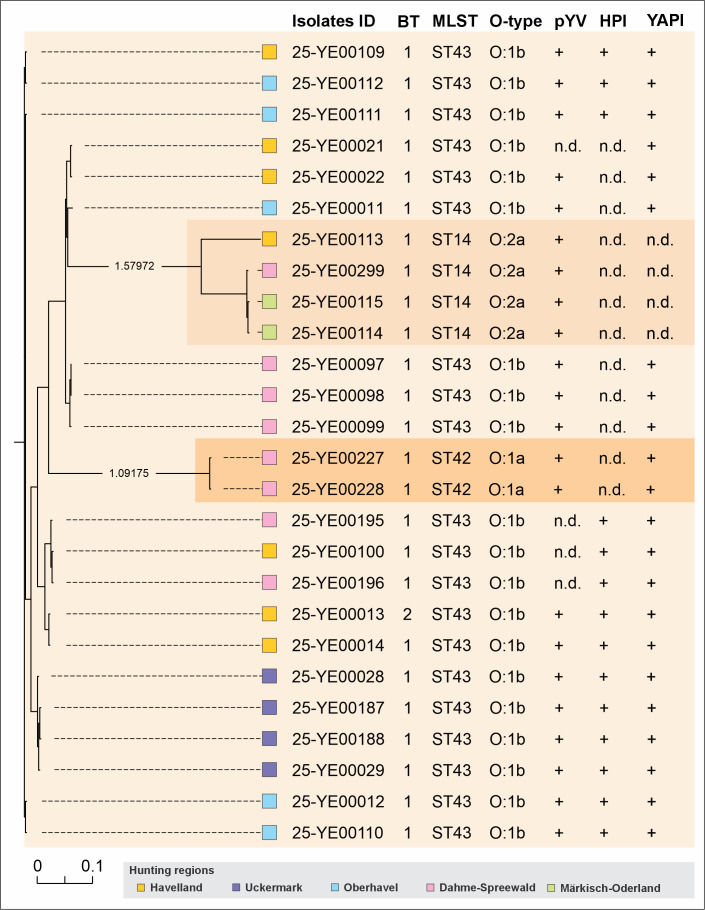
Phylogenetic relationship of *Y. pseudotuberculosis* isolates. Isolate 25-YE00007 has been used as a reference for phylogenetic comparison. All *Y. pseudotuberculosis* isolates covered 92.45% of the reference genome (genome size: 4,597,781 nt). A standard for substitution per site is given. The bioserotype, ST type, and the presence of the *Yersinia* virulence plasmid pYV and pathogenicity islands HPI and YAPI are indicated. Abbreviation: n.d., not detected.

*Yersinia pseudotuberculosis* could be recovered from 17 (21%) of the animals. All but one isolate that was melibiose negative and thus classified as BT 2 belonged to BT 1. Sequencing of 26 isolates revealed that 20 had the sero/sequence type O:1b/43, whereas 4 and 2 isolates were O:2a/14 and O:1a/42, respectively. Phylogenetically, the genomes separate into three main clusters that are consistent with the determined sequence types (Fig. 3). Most isolates (*n* = 22/26) possessed the *Yersinia* virulence plasmid pYV that may get lost during cultivation (Fig. 3 and Table S5). Other plasmids were not identified. The high-pathogenicity island (HPI), encoding proteins for the biosynthesis, regulation, and transport of the siderophore yersiniabactin, was also detected in many isolates (*n* = 14/26). However, in some isolates, the five HPI genes—*psn*, *irp1*, *irp2*, *ybtP*, and *ybtQ*—mainly involved in the yersiniabactin system, were not present (Table S5). In addition to HPI, another pathogenicity island (YAPI) containing a *pil* operon encoding a type IV pilus was detected in some isolates (*n* = 22/26). By contrast, a superantigenic toxin (YPM) existing in three variants (*ypmA*, *ypmB*, and *ypmC*) did not exist in any of them.

### *Yersinia enterocolitica* and *Yersinia pseudotuberculosis* isolates exhibited weak resistances against antibiotics

Antimicrobial susceptibility testing against 23 substances revealed mostly wild-type phenotypes of the *Y. enterocolitica* and *Y. pseudotuberculosis* isolates (Tables S6 and S7). Among *Y. enterocolitica* species, most isolates exhibited non-wild-type phenotypes against amoxicillin/clavulanic acid (AMC), erythromycin (E), and ampicillin (AMP). However, one isolate showed distinct differences in the inhibition zone diameter against cefotaxime in comparison to all other *Y. enterocolitica* of this study (Table S6). For *Y. pseudotuberculosis* isolates, a similar non-wild-type phenotype was observed against erythromycin, while sporadically, other substances (e.g., AMC and AMP) also indicated non-wild-type phenotypes (Table S7). In none of the genomes of both species acquired antimicrobial resistance determinants associated with detected antimicrobial resistances found. While in *Y. enterocolitica*, *blaA* and *vatF* were detected encoding resistances against beta-lactams and streptogramin, *Y. pseudotuberculosis* was free from any antimicrobial resistance (AMR) genes backed in the AMRFinder database. The results indicate a weak antimicrobial resistance level of pathogenic *Yersinia* species isolated from wild boars in Germany.

## DISCUSSION

The genus *Yersinia* is composed of 28 species, of which only three (*Y. enterocolitica*, *Y. pseudotuberculosis*, and *Y. pestis*) are considered to be obligatorily pathogenic for humans and animals. On the other hand, the virulence of *Y. enterocolitica*-like species (e.g., *Y. frederiksenii*, *Y. intermedia*, and *Y. kristensenii*) still needs to be clarified because they are generally regarded as non-pathogenic but have been isolated from clinical stool specimens of humans and may also contain some virulence genes like *ail*, *tccC*, and *ystB* (55–59). The same applies to *Y. enterocolitica* BT 1A strains, which are obviously diverse regarding their pathogenic potential (24, 25, 60). For that reason, all *Yersinia* isolates recovered in this study were investigated by MALDI-TOF MS. This examination disclosed a broad range of *Yersinia* species, of which up to five were isolated from a single tonsil. The dominant species was *Y. enterocolitica*, which made up more than 76% of all *Yersinia* isolates and was present in 96% of the investigated tonsils, followed by *Y. kristensenii* and *Y. pseudotuberculosis*. Thus, a fairly high number of tonsils contained both pathogenic and non-pathogenic species. While the species *Y. enterocolitica* and *Y. kristensenii* were isolated from similar numbers of tonsils in all identified hunting districts of Brandenburg, significant differences were observed for *Y. pseudotuberculosis*. However, examination of the tonsils by *wzz*-PCR did not indicate higher prevalences for this species (data not shown). The methodology used in this study was very successful for the isolation of *Yersinia*. Indeed, approximately 81% of all 1,797 colonies picked from CIN agar belonged to this genus. The long incubation for 12 weeks at 4°C prior to freezing and preparation of the tonsils obviously resulted in a strong propagation of *Yersinia*. In addition, like with minced meat (51), the filtration and centrifugation steps combined with enrichment for only 6 h helped to avoid a strong growth of other bacteria. Remarkably, after the second treatment with 0.9% KOH (end concentration 0.45%), CIN agar plates contained mainly *Yersinia* colonies. This was even more surprising, since *Y. pseudotuberculosis*, which is known to be very sensitive to KOH (43), was isolated from many tonsils. It can be suggested that during the incubation of the tonsils for 12 weeks, *Yersinia* could propagate to such high numbers that some of them survived even two KOH treatments, while most other bacteria were eliminated. To the best of our knowledge, such high *Yersinia* prevalences in wild boars have yet not been described. Other studies on this subject also report on *Y. enterocolitica* BT 1A as the predominant type, whereas pathogenic biotypes and *Y. pseudotuberculosis* have only occasionally been detected. Regrettably, there is still no cultural method specifically focusing on the isolation of pathogenic yersiniae. In the study by Reinhardt et al. (43), a preincubation for 6 h at RT prior to cold enrichment for 7 days was carried out, leading to the isolation of *Y. pseudotuberculosis* from only 10 out of 503 tonsils (2%), while 6.4% of the animals were positive by PCR. Nevertheless, the data presented here confirm the notion that wild boars are an important natural reservoir also for this species. It can, however, also not be ruled out that the long preincubation of the tonsils for 12 weeks preferentially promoted the growth of *Y. enterocolitica* BT 1A, which may have suppressed other biotypes, resulting in their underrepresentation.

As almost all *Y. enterocolitica* isolates belonged to BT 1A, a selection of them was sequenced. The analysis revealed a high diversity of STs. What they had in common was the content of certain virulence genes. All isolates contained the toxin genes *yaxA*, *yaxB*, and *ystB*, the primary internalization factor gene *invA*, the gene *hreP* for the bacterial subtilisin/Kexin-like protease, and the *fep-fes* gene cluster for siderophore uptake. The regulator gene *ymoA* was also detected in all isolates. Moreover, a number of isolates additionally contained the virulence genes *ail*, *myfA*, and *tccC*, suggesting that they may have a pathogenic potential. However, there was no close link to specific STs. Thus, it remains open if certain STs are more virulent than others. Many BT 1A isolates harbor plasmids, similar to those of other *Yersinia* species or related genera like *Escherichia* and *Salmonella*. The fact that some of the related plasmids are apparently conjugative suggests extensive transfer between these bacteria. By contrast, the two highly pathogenic bio/serotype 1B/O:8 isolates contained pYV, which normally does not occur in BT 1A.

Unlike *Y. enterocolitica* BT 1A, the *Y. pseudotuberculosis* isolates of this study were much more similar to each other. Almost all of them belonged to BT 1 and were allocated to only three sero/sequence types (O:1a/42, O:1b/43, and O:2a/14), of which O:1b/43 was predominant. In a former study on the presence of *Y. pseudotuberculosis* in tonsils of wild boars that was also conducted in Northeast Germany, 7 out of 10 isolates belonged to ST42 with the biotype 1 or 2 and the serotype O:1a or O:1b (42, 43). This suggests that wild boars in this region may contain various *Y. pseudotuberculosis* types. On the other hand, as with the isolates in this study, two sero/sequence type O:1a/42 strains, one O:1b/43 strain, and two O:2a/14 strains, all of which belonged to BT 1, had been isolated in a German zoo from a diseased Patagonian mara, a short-eared elephant shrew, and pudus, respectively (61). Interestingly, in both studies, all O:1a/42 and O:1b/43 isolates contained the complete HPI, while the high pathogenicity island was lacking in O:2a/14. It is well known that the HPI is a mobile element, which can be transmitted between strains (62). Some O:1b/43 isolates in this study additionally contained the pathogenicity island YAPI encoding a type IV pilus that contributes to pathogenicity (63).

Besides assessment of pathogenicity, the surveillance of antimicrobial resistance in bacteria from wildlife populations provides an overview of environmental health and pollution in ecosystems. In contrast to other *Enterobacterales* such as *E. coli* and *Klebsiella* species, limited information about antimicrobial resistance in *Yersinia* is available. A recent systematic review indicated a high prevalence of some antimicrobial resistances, including ampicillin, amoxicillin, erythromycin, and sulfonamides, while others were less prevalent (64). Overall, the results presented here indicate weak antimicrobial resistance in pathogenic *Yersinia* species isolated from wild boars in Germany.

### Conclusion

The study suggests that a high proportion of wild boars in Northeast Germany contain pathogenic and non-pathogenic *Yersinia* species. While the long cold enrichment of the tonsils for 12 weeks used here is certainly not applicable for routine diagnostics, it helped demonstrate that *Yersinia* is widespread in these animals. Thus, it can be expected that wild boars play a major role in the dissemination of the bacteria in the environment. Moreover, it is conceivable that pathogenic species like *Y. pseudotuberculosis* may contaminate vegetables (e.g., carrots), leading to outbreaks, as already reported in Scandinavia.

## MATERIALS AND METHODS

### Sampling, isolation, and cultivation of *Yersinia*

Game samples were taken during hunts in the 2023/2024 season. Sampling was conducted in certain districts of different counties in the German federal state of Brandenburg. Animals were legally hunted for human consumption and game management. The age of the animals was assigned to three groups (0 [juveniles]: <1 year, 1 [yearlings]: 1–2 years, and 2 [adults]: >2 years old) by experienced hunters as previously described (e.g., tooth and physical appearance) (65). Samples were taken by hunters and employees of the German Federal Forestry Service from the animals during evisceration. They were collected in sterile, sealable cups and transported within 12 h in a cool box to the German Federal Institute for Risk Assessment for storage at 4°C and subsequent analysis.

Tonsils of 81 wild boars were collected between November 2023 and January 2024. The animals were shot in six different counties of Brandenburg (Fig. 1A) on driven community and individual hunts. The age, sex, and origin of the animals are summarized in Table S1 . The samples were provided to the “Consiliary Laboratory for *Yersinia* in Food” at the German Federal Institute for Risk Assessment, stored at 4°C for 12 weeks, and then frozen at −80°C until usage. After thawing, samples were homogenized in 225 mL peptone-sorbitol-bile salt (PSB) broth using a Bagmixer (Interscience, Mourjou, France) for 4 min at level 2. One hundred microliters of each sample was directly plated on a cefsulodin-irgasan-novobiocin (CIN) agar and incubated at 28°C overnight, while 500 μL was mixed with 500 μL KOH (0.9%, 0.45%, and 0.225%) for 20 s, followed by plating of 250 μL on CIN and incubation, as stated above. The rest of each sample was filtrated as previously described (51). Thereafter, one-half of the filtrate was centrifuged, while the other half was centrifuged after incubation at 28°C for 6 h (51). Pellets were resuspended in 450 μL PBS. One hundred fifty microliters of each cell suspension was mixed with 150 μL KOH (0.9%, 0.45%, and 0.225%) and incubated for 20 s. One hundred microliters of each preparation was plated on CIN agar. In cases where a bacterial lawn was obtained after incubation overnight at 28°C, the lawn was removed by adding 1.5 mL PSB to the agar plate and resuspending the cells using a Drigalski spatula. The concentrated cell suspension was subjected to a 10-fold serial dilution in PBS buffer up to a 10^−7^ dilution. Five hundred microliters of the 10^−3^, 10^−4^, 10^−5^, 10^−6^, and 10^−7^ dilutions was then mixed with 500 μL KOH (0.9%) and incubated for 20 s, and 100 μL of the mixture was plated on CIN, followed by incubation, as described above. For propagation, all *Yersinia* isolates of this study were grown in lysogeny broth (66).

### Detection and typing of *Yersinia* colonies

Colonies on CIN agar that showed the typical “bull’s-eye” morphology were subjected to MALDI-TOF MS. Each colony was analyzed twice using the direct transfer method with HCCA matrix on a Microflex LT Biotyper (Bruker Daltonics GmbH & Co. KG, Bremen, Germany) as recommended. Mass spectra were collected using the software flexControl (v3.4) and MBT Compass HT. To discriminate pathogenic BTs from BT 1A, all *Y. enterocolitica* isolates were analyzed by multiplex PCR using primers for the genes *ail*, *gluF*, *ystA*, and *ystB* (52). Pathogenic isolates were typed by the biotyping scheme established by Wauters et al. (67). The serotype of those *Y. enterocolitica* isolates was determined by PCR using primers for O:3, O:5.27, O:8, and O:9 (68–70).

### Whole-genome sequencing of isolates and bioinformatic analyses

To characterize and compare isolates by bioinformatic analyses of WGS data, at least one *Y. enterocolitica* and one *Y. pseudotuberculosis* isolate from each positive tonsil were subjected to paired-end short-read sequencing. Genomic DNA was extracted from 1 mL bacterial culture using the PureLink DNA extraction kit (Thermo Fisher Scientific, Germany) according to the manufacturer’s recommendations. Illumina sequencing was conducted on a NextSeq 500 device using a sequencing library prepared with the Nextera DNA Flex Kit in a 2 × 150 bp cycle run with the NextSeq 500/550 Midoutput Kit (v2.5) (Illumina, San Diego, CA, USA). Raw reads were subjected to the Aquamis pipeline (https://gitlab.com/bfr_bioinformatics/AQUAMIS) (v1.4.1) using fastp (read trimming/read quality check [QC]), shovill (*de novo* assembly), mash (reference search/species determination), QUAST (v5) (assembly QC), confindr (inter-/intragenus contamination analysis), and kraken2 (read/assembly-based taxonomic profiling) (71). *In silico* typing of the genomes was conducted using the Bakcharac pipeline (https://gitlab.com/bfr_bioinformatics/bakcharak). Genome annotation was performed using the Prokaryotic Genome Annotation Pipeline (v4.11) implemented in NCBI (72). The serotype of *Y. pseudotuberculosis* isolates was determined by sequence analysis of the O-antigen gene cluster, as previously described (73). Phylogenetic analysis (SNP-based) was conducted using CSI phylogeny (v1.4) with default parameters.

### Genome accession

The nucleotide sequences of *Y. enterocolitica* (*n* = 85) and *Y. pseudotuberculosis* isolates (*n* = 26) were submitted to GenBank (NCBI) under BioProject PRJNA1392632. Accession numbers for individual genome data sets are given in .

### Antimicrobial susceptibility testing

Agar disk diffusion assays with cation-adjusted Mueller–Hinton medium were performed for evaluation of phenotypic AMRs according to the standards recommended by the Clinical and Laboratory Standards Institute (74). The assays were performed at 28 ± 1°C for 24–28 h. Antibiotic disks (Thermo Fisher, East Grinstead, West Sussex, UK) were used as specified: amikacin (30 µg), AMC (30 µg), AMP (10 µg), cefepime (30 µg), cefotaxime (30 µg), ceftazidime (10 and 30 µg), chloramphenicol (30 µg), ciprofloxacin (5 µg), enrofloxacin (5 μg), E (15 µg), florfenicol (30 µg), gentamicin (10 µg), imipenem (10 µg), meropenem (10 µg), nalidixic acid (30 µg), norfloxacin (10 µg), oxolinic acid (2 µg), oxytetracycline disk (30 µg), streptomycin (10 µg), sulfonamide (300 µg), tetracycline (30 µg), trimethoprim (5 µg), and trimethoprim/SMX (25 µg). The *Escherichia coli* strain ATCC 25922 was used as quality control for antimicrobial resistance determination (74).

## Data Availability

Accession numbers for genomes determined in this study are available at GenBank (NCBI) under BioProject PRJNA1392632.

## SUPPLEMENTAL MATERIAL

Fig S1 spectrum.00934-26-s0001.tif Multiplex-PCR analysis of *Y. enterocolitica* isolates.

Supplemental tables spectrum.00934-26-s0002.xlsx Tables S1 to S8

Supplemental legend spectrum.00934-26-s0003.docx Descriptive legend for Fig. S1.

ASM does not own the copyrights to Supplemental Material that may be linked to, or accessed through, an article. The authors have granted ASM a non-exclusive, world-wide license to publish the Supplemental Material files. Please contact the corresponding author directly for reuse.
