# Supplementary material for: High Yersinia prevalence in tonsils of wild boars hunted in Northeast Germany using a novel protocol including long cold enrichment
Source: Microbiol Spectr. 2026 Jul 2;14(8):e00934-26. doi: 10.1128/spectrum.00934-26 (PMC13435784; doi:10.1128/spectrum.00934-26)
Supplement: Supplementary file 1 [file spectrum.00934-26-s0003.docx]

Figure S1. Multiplex-PCR analysis of *Y. enterocolitica* isolates. A: lanes 1 and 15, marker, lanes 2 to 11, single BT 1A isolates, lane 12, bioserotype B4/O:3 control (*ail*+/*ystA*+), lane 13 BT 1A control (*gluF*+/*ystB*+), lane 14, water. B: lane 1, marker, lanes 2 to 16, single BT 1A isolates, of which isolate 8 and 11 are *ail*+, lane 17, bioserotype B4/O:3 control (*ail*+/*ystA*+), lane 18 BT 1A control (*gluF*+/*ystB*+), lane 19, water.
